# Prevalence of Hepatitis B Virus Infection in Tanzania: A Systematic Review and Meta-Analysis

**DOI:** 10.1155/2024/4178240

**Published:** 2024-06-05

**Authors:** Semvua B. Kilonzo, Igembe Nkandala, Ladius Rudovick, Hyasinta M. Jaka, Mariam M. Mirambo, Stephen E. Mshana, Violet D. Kajogoo, Elichilia R. Shao

**Affiliations:** ^1^Internal Medicine Department, Catholic University of Health and Allied Sciences, P.O. Box 1464, Mwanza, Tanzania; ^2^Department of Microbiology and Immunology, Catholic University of Health and Allied Sciences, P.O. Box 1464, Mwanza, Tanzania; ^3^Department of Clinical Trials, Tanzania Diabetes Association, P.O. Box 65201, Dar es salaam, Tanzania; ^4^Internal Medicine Department, Kilimanjaro Christian Medical University College, P.O. Box 2240, Moshi, Tanzania

## Abstract

**Methods:**

We systematically searched the PubMed, Web of Science, African Journals Online, Embase, Cochrane Library, and Google Scholar databases for studies conducted up to March 1, 2023, that estimated the prevalence of HBV in Tanzania based on HBV surface antigen measurements. The DerSimonian–Laird random effects model was used to estimate the overall prevalence of HBV with 95% confidence intervals (CIs). Potential sources of heterogeneity were also investigated.

**Results:**

Thirty-one studies with a total sample size of 37,988 were included in the meta-analysis. The overall average HBV prevalence estimate in Tanzania was 6.91% (95% CI = 5.18–8.86%). Subgroup analysis revealed the highest prevalence in the northern zone (9.32%, 95% CI; 2.24–20.36%), among the blood donors (18.72%, 95% CI: 17.43–20.05%) and among the community volunteers (8.76%, 95% CI: 4.55–14.15%). The lowest prevalence was observed in the lake zone at 4.66% (95% CI: 3.49–5.99) and in pregnant women at 4.72% (95% CI: 3.42–6.21). The overall between-study variability showed significant heterogeneity (*I*^2^ = 97.41%, *P* < 0.001).

**Conclusions:**

Our results showed that Tanzania is a country with moderately high HBV endemicity, with large interregional differences and significantly high numbers of HBV infections within the community. This underscores the need for immediate development of targeted prevention strategies and further epidemiological studies to better understand the pattern of the disease.

## 1. Introduction

Hepatitis B virus (HBV) infection poses a significant global public health burden, with approximately 296 million people living with chronic hepatitis B (CHB) worldwide [1]. HBV infection can lead to serious liver diseases, such as cirrhosis and liver cancer. In sub-Saharan Africa, including Tanzania, the prevalence of HBV infection is particularly high. Available subpopulation studies in different parts of Tanzania have shown the prevalence of HBV to be 1.2–11.2% [2].

Despite the significant global burden associated with HBV infection, recent estimates indicate that only approximately 10% of people living with this disease have been formally diagnosed, and only 13% of those diagnosed are receiving treatment [3]. This global estimate coincides with the local data from Tanzania, where less than 10% of the population was screened for HBV, with minimal detection and treatment rates [4]. This concerning gap in diagnosis and subsequent treatment exposes a substantial number of individuals to an increased lifetime risk of liver-related complications, such as cirrhosis, hepatocellular carcinoma (HCC), and events of end-stage liver disease and death. Indeed, more than 66% of cases in the country with HCC were correlated with HBV infection [5].

Globally, there have been several efforts to address this issue, with the goal of eliminating HBV as a public health threat by 2030 [6]. Prevention through vaccination and antiviral treatment is a key strategy for achieving this goal. However, according to the recent report from the World Health Organization (WHO) [7], this target is likely to be missed, particularly in low- and middle-income countries, mainly due to a lack of comprehensive viral hepatitis prevention and control programs and poor surveillance systems for the disease. According to this report, there is still a high number of new infections and deaths; 1.5 million people were newly infected with HBV in 2019, with 1.1 million deaths.

In response to the global HBV elimination goal, Tanzania launched a national strategic plan for viral hepatitis in 2018 [4]. However, the effective policy implementation is still limited, as vaccination rates for high-risk groups have remained below the desired level, 2% in pregnant women [8], 74% in healthcare workers (HCWs) [9], and 0% in people living with HIV (PLWHIV) [2]. The WHO [6] recommends the HBV vaccination for all individuals at high risk of HBV infection. Additionally, the number of eligible patients receiving antiviral treatment for HBV is limited in the country [10]. The inconsistency of the data, coupled with insufficient surveillance systems in the country, may be the major factor hindering effective policy implementation, subsequent preventive strategies, and resource allocation. To address this issue, a systematic review and meta-analysis of available studies in Tanzania were conducted to estimate the prevalence of CHB in the country, describing the disease pattern.

## 2. Materials and Methods

### 2.1. Search Strategy

This study was registered with the International Prospective Register of Systematic Reviews (PROSPERO) with registration number CRD42023472128 and followed the Preferred Reporting Items for Systematic Reviews and Meta-Analyses (PRISMA) guidelines [11]. A systematic literature search was conducted in the following electronic databases: PubMed, Web of Science, African Journals Online, Embase, Cochrane Library, and Google Scholar, for studies up to March 2023. For PubMed, the following MeSH terms and texts were used: “Hepatitis B” [MeSH] OR “HBV” [MeSH] OR “Hepatitis B prevalence” [MeSH] OR “Viral hepatitis” [MeSH] AND “Hepatitis B virus” [MeSH] AND “Hepatitis B Tanzania” [MeSH] OR “Hepatitis B Mwanza” [MeSH] OR “Hepatitis B Kilimanjaro” [MeSH] OR “Hepatitis B Dar es salaam” [MeSH] OR “Hepatitis B Mbeya” [MeSH] OR “Hepatitis B Morogoro” [MeSH] OR “Hepatitis B Iringa” [MeSH] OR “Hepatitis B Pwani” [MeSH] OR “Hepatitis B Zanzibar” [MeSH] OR “Hepatitis B Kagera” [MeSH] OR “Hepatitis B Arusha” [MeSH] OR “Hepatitis B Manyara” [MeSH] OR “Hepatitis B Njombe” [MeSH] OR “Hepatitis B Katavi” [MeSH] OR “Hepatitis B Tanga” [MeSH] OR “Hepatitis B Ruvuma” [MeSH] OR “Hepatitis B Singida” [MeSH] OR “Hepatitis B Shinyanga” [MeSH] OR “Hepatitis B Simiyu” [MeSH] OR “Hepatitis B Geita” [MeSH] OR “Hepatitis B Mara” [MeSH] OR “Hepatitis B Rukwa” [MeSH] OR “Hepatitis B Mtwara” [MeSH] OR “Hepatitis B Kigoma” [MeSH] OR “Hepatitis B Lindi” [MeSH] OR “Hepatitis B Tabora” [MeSH] OR “Hepatitis B Pemba” [MeSH] OR “Hepatitis B Dodoma” [MeSH]. The latter is a list of all administrative regions in Tanzania. Two independent authors (SBK and VDK) screened the titles and abstracts of the manuscripts to determine their relevance, and the full texts of the selected studies were subsequently reviewed. Similar search strategies were used for other databases that included terms such as hepatitis B virus, viral infections, prevalence, Tanzania, and prevalence of hepatitis in the various target groups.

### 2.2. Eligibility

#### 2.2.1. Inclusion Criteria

The inclusion criteria were studies conducted in Tanzania, published in English before March 2023, used the hepatitis B surface antigen (HBsAg) test for diagnosing HBV infection, and included participants of any age.

#### 2.2.2. Exclusion Criteria

Case reports, reviews, preprints, and studies with insufficient or inaccessible data were excluded.

### 2.3. Data Collection and Management

Mendeley software was used as the reference manager to remove duplicates of the studies that were identified from the electronic databases and to generate bibliographies. The titles and abstracts that were produced from electronic databases were independently screened by two authors as per eligibility criteria. The full texts of the qualified studies were then reviewed, and the data were extracted and entered into a Microsoft Excel-designed extraction form using the following variables: publication year, study design, study location (regional/zone), population (general, PLWHIV, pregnant women, HCW, people who inject drugs (PWID), and children), study year, sample size, prevalence of HBV, and diagnosis method. A third reviewer was involved in double-checking the correctness of the data entry and resolving disagreements.

### 2.4. Quality Assessment

Quality assessment was done using the Newcastle-Ottawa Quality Assessment Scale [12]. All the parameters of this scale (standardized methods for confirming diagnosis, large sample size, multicenter study, appropriate statistical methods that report results, accounting for confounders, clear methodology of selection of participants, and representativeness of the population) were considered in our meta-analysis. The quality assessment was carried out by two independent reviewers, and disagreements were resolved by a third reviewer.

### 2.5. Statistical Analysis

Quantitative data on the overall number of subjects with HBV were extracted, as were the data in the subgroups. The HBV prevalence across studies was calculated, and due to heterogeneity, the data are presented as ranges of values with 95% confidence interval (CI). Furthermore, weighted means were calculated considering the different study sizes.

For the meta-analysis, contingency tables were created in an Excel spreadsheet to compare the prevalence of HBV in different study populations. Given the inherent variability among observational studies, we used the random effects model of DerSimonian and Laird to estimate the HBV prevalence in the included studies, as used elsewhere [13]. Heterogeneity across studies was assessed using the heterogeneity index (*I*^2^); *I*^2^ > 70% suggested high heterogeneity, 50–69 indicated substantial heterogeneity, and <49 indicated low heterogeneity.

## 3. Results

### 3.1. Study Identification

**Figure 1:**
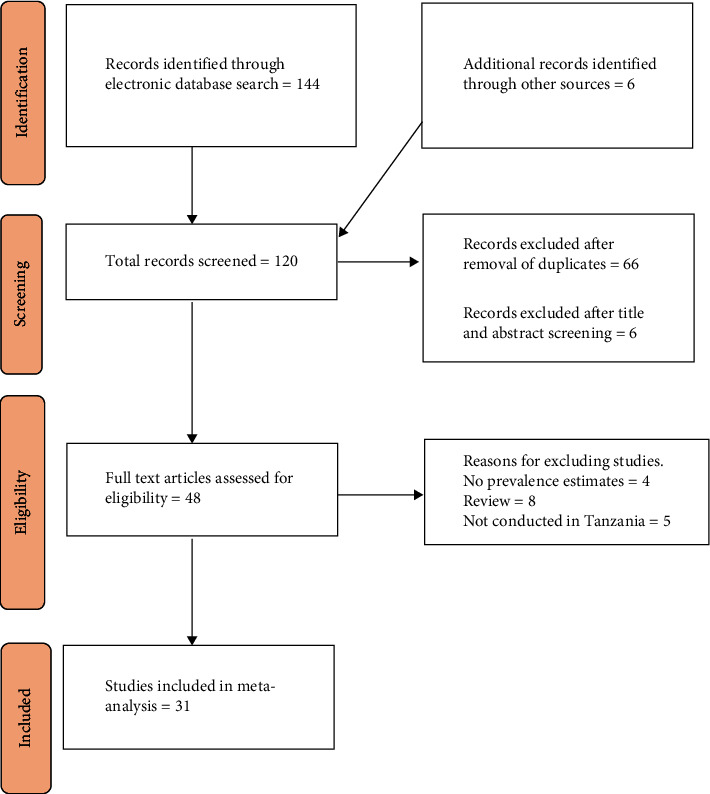
PRISMA flow diagram for identification and selection of articles for inclusion in the review.

The flowchart for the selection of studies is presented in Figure 1. A total of 120 studies were found in various electronic databases. After removing duplicates, 66 articles remained. After screening the titles and abstracts, 6 more studies were removed and 48 studies were chosen for full manuscript reading. From those, 17 studies were excluded due to missing prevalence (4), conducted outside Tanzania (5), and 8 being reviews leaving 31 studies for the final review. The risk of bias assessment according to the Newcastle-Ottawa Quality Assessment Scale is shown in Supplementary Table 1.

### 3.2. Study Characteristics

We included all studies that met the eligibility criteria and were conducted before 01 March 2023 in our review.  Table 1 shows the characteristics of the studies that were eligible for our meta-analysis. A total of 31 studies were analyzed, with a combined sample size of 37,988. The duration of the studies ranged from two months to nine years, with the majority being cross-sectional studies (26 out of 31; 83.9%). Approximately one-third of the studies (11 out of 31; 35.5%) were conducted in the eastern zone of the country, specifically in the Dar es Salaam and Morogoro regions. The Zanzibar, southern, and central zones had minimal representation, with only one study (3.2%) each. Among the reviewed studies, Hawkins et al. [31] had the largest sample size (17,539), while Machange et al. [23] had the smallest sample size (68). The majority of the studies (17 out of 31; 54.8%) were conducted between 2013 and 2023, while only 5 out of 31 (16.1%) were conducted between 1991 and 2001. Rapid diagnostic tests (RDTs) were the most commonly used method for diagnosing HBV in 17 (54.8%) studies.

### 3.3. Prevalence of HBV Infection in Tanzania

**Figure 2:**
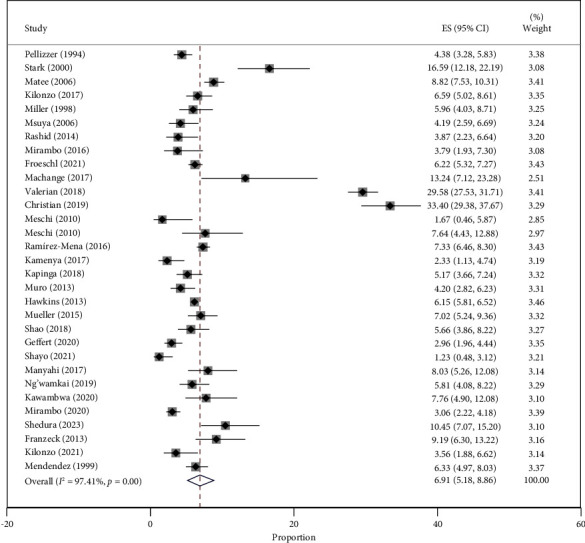
Forest plot of the studies on HBV prevalence in Tanzania published from 1994 to 2023.

The combined prevalence of HBV among the 37,988 participants was 6.91% (95% CI = 5.18–8.86%), with a high level of heterogeneity at 97.41% (*P* < 0.001). The prevalence varied significantly across the included studies, ranging from 1.23% (95% CI = 0.48–3.12%) in individuals with sickle cell disease to 33.40% (95% CI = 29.38–37.67%) in PLWHIV (Figure 2).

### 3.4. Subgroup Analysis of HBV Prevalence

**Figure 3:**
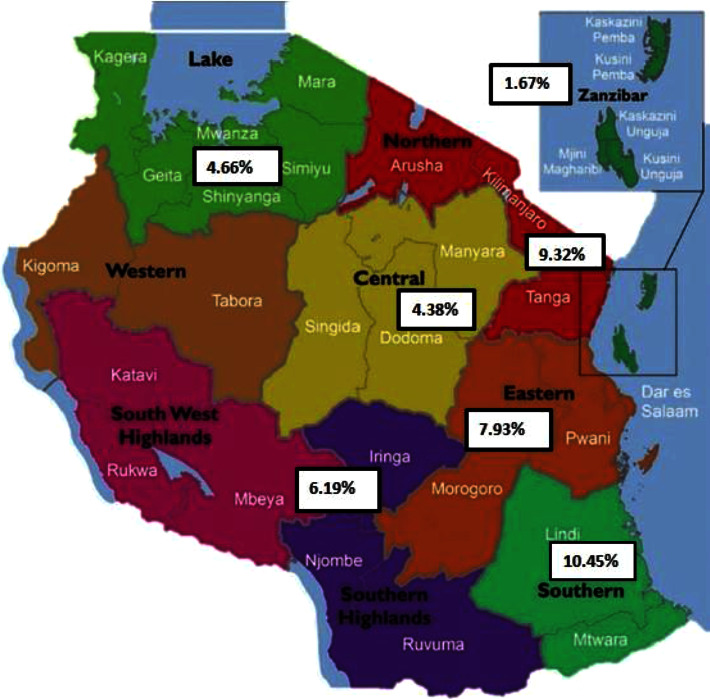
Map of Tanzania showing the prevalence of hepatitis B infection in different geographical zones of the country. Modified map adopted from Suleiman [43]. Tanzania regions are classified into 9 zones: (1) Eastern Zone (Morogoro, Pwani, and Dar es Salaam); (2) Northern Zone (Arusha, Kilimanjaro, and Tanga); (3) Lake Zone (Kagera, Mwanza, Simiyu, Mara, Shinyanga, and Geita); (4) Western Zone (Kigoma and Tabora); (5) Central Zone (Dodoma, Manyara, and Singida); (6) Southern Highlands (Katavi, Mbeya, Rukwa, Iringa, and Njombe); (7, 8) Southern Zone (Lindi, Ruvuma, and Mtwara); and (9) Zanzibar Zone.

As shown in Table 2, our meta-analysis divided the studies into groups based on geographical location, year of study, studied population (PLWHIV, blood donors, HCW, pregnant women, and PWID), and HBV diagnosis method. When considering the geographical location of the country, the highest prevalence of HBV was found in the northern zone, with 9.32% (95% CI; 2.24–20.36%) and a total of 3779 participants. The eastern zone had the next highest prevalence of 7.93% (95% CI; 5.57–10.66%) with a sample size of 25,487. The lowest prevalence of 4.66% (3.49–5.99) was observed in the lake zone. The southern, central, and Zanzibar zones were each represented by one study (Figure 3).

Recent studies conducted from 2013–2023 (sample size of 11,503) reported the highest pooled HBV prevalence of 7.48% (95% CI = 3.96–11.97%) compared to older studies. Among the two HBV diagnostic methods used, studies that used enzyme-linked immunosorbent assay (ELISA) (sample size 8457) reported a higher HBV prevalence of 7.79% (95% CI; 4.08–12.53) compared to studies that used RDT (6.24%, 95% CI; 4.65 – 6.62%). Additionally, HBV prevalence was highest in the general population (blood donors and community volunteers) (18.72%, 95% CI: 17.43–20.05%) and (8.76%, 95% CI: 4.55–14.15%), respectively, compared to high-risk groups such as PLWHIV (7.31%, 95% CI: 5.22–9.72%), HCW (7.06%, 95% CI: 4.63–9.93%), and PWID (5.32%, 95% CI: 3.44–7.57). Pregnant women had the lowest prevalence of 4.72% (95% CI: 3.42–6.21%).

## 4. Discussion

Effective policy implementation requires data on the magnitude of disease. This meta-analysis review reports the prevalence of HBV in different populations in Tanzania from 1994 to 2023. Our findings showed a pooled prevalence of 6.91%, with variations observed among different geographical zones, study years, diagnostic methods, and population subgroups. This prevalence is similar to that reported in Ethiopia (6.0%) [13] and the average prevalence in four other East African countries (6.03%). Tanzania had a lower prevalence of 5.16% compared to Kenya and Uganda, which had higher prevalence of 8.54% and 8.45%, respectively [44]. Similarly, a neighboring country, Malawi, had a higher prevalence of 8.1% [45]. These results suggest that Tanzania has intermediate-high endemicity of HBV, while most neighboring countries have high endemicity according to the WHO classification [46]. The lower prevalence in Tanzania may be attributed to the government's efforts to improve preventive measures, such as early infant HBV vaccination. Tanzania has higher cumulative vaccination coverage rates (89.6%) compared to Uganda (77.6%) and Kenya (86.7%) [47]. Other factors, including HBV genotypes and high-risk behaviors, may also contribute to these differences. Nonetheless, Tanzania being intermediate-high HBV endemicity, more aggressive public health measures are needed in the country to control HBV and align with global targets for elimination by 2030.

Our research found significant differences in the occurrence of HBV in different areas of the country. Studies in the northern and eastern regions reported higher rates than the national average, while lower rates were reported in the lake and southern highlands regions. These findings suggest that there may be variations in the factors that contribute to HBV infection, such as prenatal screening, vaccination rates, community awareness, and the persistence of the infection. However, further investigation is necessary to understand the underlying reasons for this pattern. Similar disparities in HBV prevalence have been reported in other countries, including Iran [48], China [49], and Kenya [50], independent of factors like the studied population or diagnostic methods used. As for the southern, Zanzibar, and central regions, it is challenging to determine the overall prevalence of HBV due to the limited number of studies conducted in each area, with different populations and timeframes. In the southern region, Shedura et al. [39] analyzed the prevalence of HBV among pregnant women attending antenatal clinics in 2022. In Zanzibar [26], febrile outpatients were analyzed in 2007 while the analyzed population in the central region included all outpatients attending districts and regional hospitals from 1991 to 1992 [14].

Our study also assessed the prevalence of HBV infection in different populations, and one significant finding was the higher prevalence of HBV in the general population (blood donors and community volunteers) compared to high-risk groups such as PLWHIV, HCW, PWID, and pregnant women. This contrasts with findings from other studies in intermediate- and high-endemic areas. For example, in Bangladesh, the national average prevalence of HBV in the general population is 4%, lower than that in PWID (7.5%) and HCW (7.3%) [51]. Similarly, in China, a recent meta-analysis reported a national HBV prevalence of 3.8% in the general population, with higher rates in high-risk populations such as PLWHIV (10.7%) and PWID (15.0%) [52]. Another study in Sierra Leone found a higher prevalence of 15.9% among PLWHIV compared to the national average of 13.0%, while pregnant women and HCW had lower percentages of 9.7% and 11.9%, respectively [53]. One possible explanation for our findings is that the high-risk populations consistently have access to healthcare facilities, making them more likely to receive health education and vaccination against HBV infection compared to the general population. Previous studies in Tanzania have shown that more than two-thirds of HCW were vaccinated against HBV infection, and general knowledge about HBV was good [54, 55]. These findings were backed up by another study by Ndunguru et al. [56] that further revealed that knowledge and HBV vaccine uptake among the HCW were significantly higher in the urban areas compared to rural areas. However, even though the prevalence in high-risk populations is lower than the community, it is still higher than the national average as found in our study. Therefore, these findings highlight the importance of public health measures and call for simultaneous efforts to strengthen HBV elimination programs both in the community and healthcare settings.

In terms of the diagnosis methods, the prevalence rate of HBV detected using RDT was found to be lower than that of studies using ELISA. This may be due to the lower sensitivity of RDT compared to ELISA, with reported sensitivities of 70.0% and 78.0% in Togo and Gabon, respectively [57, 58]. The performance of RDT can also be influenced by factors such low levels of HBsAg and HBV deoxyribonucleic acid (DNA), HBV genotypes, specific brand of HBsAg tests used, and the HIV status of the individual [59]. Due to these shortcomings, the WHO recommends using RDT only in the settings where laboratory testing is limited and/or in populations where access to rapid testing would facilitate linkage to care and treatment [60]. In this meta-analysis, the majority (54.8%) of published studies, including those involving PLWHIV, used RDT as a diagnostic method, which may have led to an underestimation of the true prevalence of HBV in our study.

### 4.1. Strengths

This study employed an extensive search approach across significant data sources and included a large number of studies and various subgroup populations from most geographical zones of the country. Consequently, our findings provide an accurate representation of the current HBV situation in Tanzania.

### 4.2. Limitations

One significant drawback of this meta-analysis was the uneven representation of certain subgroups such as children and various geographical zones. Only one study [30] focused on the population of infants who are particularly susceptible to HBV infection through maternal transmission. Similarly, only one study was available for some geographical zones such as southern [39], central [14], and Zanzibar [26]. Therefore, it is important to be cautious when interpreting the overall findings of our study.

## 5. Conclusion

To the best of our understanding, this study is the first to provide a meta-analysis on the prevalence of HBV in Tanzania. The current literature suggests that Tanzania is a country with an intermediate-high endemicity of HBV, but with significant variations between regions. Additionally, this study confirms a considerable number of HBV infections within the community. These results highlight the immediate need for targeted prevention strategies, such as awareness programs, universal immunization, and relevant policies. Moreover, further research should be conducted to better understand the underlying factors influencing the observed infection patterns.

## Figures and Tables

**Table 1:** Characteristics of the included studies in the systematic review and meta-analysis for the prevalence of hepatitis B in Tanzania from 1994 to 2023.

Study author	Publication year	Study design	City	Population type	Study year	Sample size	Prevalence (%)	Method
Pellizzer et al. [14]	1994	Cross-sectional	Dodoma	Outpatients	1991-1992	1004	4.4	ELISA
Stark et al. [15]	2000	Cross-sectional	Kilimanjaro	Community	1996	211	16.6	ELISA
Matee et al. [16]	2006	Cross-sectional	Dar es salaam	Blood donors	2004-2005	1599	8.8	ELISA
Kilonzo et al. [17]	2017	Cross-sectional	Mwanza	PLWHIV	2014-2015	743	6.6	RDT
Miller et al. [18]	1998	Cross-sectional	Dar es salaam	Community	1992	403	6	ELISA
Msuya et al. [19]	2006	Cross-sectional	Kilimanjaro	Outpatients	1999	382	4.2	ELISA
Rashid et al. [20]	2014	Cross-sectional	Dar es salaam	Pregnant women	2010	310	3.9	ELISA
Mirambo et al. [21]	2016	Cross-sectional	Mwanza	Pregnant women	2014	211	3.8	RDT
Froeschl et al. [22]	2021	Cohort	Mbeya	Community	2002–2010	2363	5	RDT
Machange et al. [23]	2017	Cross-sectional	Kilimanjaro	HCW	2014	68	13.2	ELISA
Valerian et al. [24]	2018	Cross-sectional	Kilimanjaro	Blood donors	2016	1829	29.6	ELISA
Christian et al. [25]	2019	Cohort	Dar es salaam	PLWHIV	2014-2015	494	33.4	RDT
Meschi et al. [26]	2010	Cross-sectional	Zanzibar	Outpatients	2007	120	1.7	ELISA
Meschi et al. [26]	2010	Cross-sectional	Iringa	Outpatients	2007	157	6.4	ELISA
Ramírez-Mena et al. [27]	2016	Cohort	Morogoro	PLWHIV	2005–2015	3097	7.3	RDT
Kamenya et al. [28]	2017	Cross-sectional	Kilimanjaro	PLWHIV	2015	300	2.3	RDT
Kapinga and Aboud [29]	2018	Cross-sectional	Kagera	Pregnant women	2017	600	7.2	RDT
Muro et al. [30]	2013	Cross-sectional	Kilimanjaro	Children	2006–2009	547	4.2	ELISA
Hawkins et al. [31]	2013	Cohort	Dar es salaam	PLWHIV	2004–2011	17539	6.2	RDT
Mueller et al. [32]	2015	Cross-sectional	Mwanza	HCW	2012	598	7.2	ELISA
Shao et al. [33]	2018	Cross-sectional	Kilimanjaro	HCW	2015-2016	442	5.7	RDT
Geffert et al. [8]	2020	Cross-sectional	Mwanza	Pregnant women	2014-2015	743	3	RDT
Shayo et al. [34]	2021	Cross-sectional	Dar es salaam	SCD patients	2018-2019	325	1.2	RDT
Manyahi et al. [35]	2017	Cross-sectional	Dar es salaam	Pregnant women	2014	249	8	ELISA
Ng'wamkai et al. [36]	2019	Cross-sectional	Mwanza	Pregnant women	2018	499	5.8	RDT
Kawambwa et al. [37]	2020	Cross-sectional	Dar es salaam	PWID	2017	219	7.8	RDT
Mirambo et al. [38]	2020	Cross-sectional	Mwanza	Students	2016	1211	3.1	RDT
Shedura et al. [39]	2023	Cross-sectional	Mtwara	PLWHIV	2022	220	10	RDT
Franzeck et al. [40]	2013	Prospective	Morogoro	PLWHIV	2011-2012	272	9.2	RDT
Kilonzo et al. [41]	2021	Cross-sectional	Mwanza	PWID	2019-2020	253	3.6	RDT
Menendez et al. [42]	1999	Cross-sectional	Morogoro	Pregnant women	1995	980	6.3	ELISA

ELISA: enzyme-linked immunosorbent assay; PLWHIV: people living with HIV; HCW: healthcare worker; RDT: rapid diagnostic test; PWID: people who inject drugs.

**Table 2:** Subgroup analysis of the estimated prevalence of HBV in Tanzania in the studies published from 1994 to 2023.

Variable	Number of studies	Sample size	Prevalence % (95% CI)	Heterogeneity (%)	*P* value
Geographical zone					
Southern	1	220	10.45 (7.07–15.20)	NA	NA
Northern	7	3779	9.32 (2.24–20.36)	98.74	<0.001
Eastern	11	25487	7.93 (5.57–10.66)	96.73	<0.001
Southern highlands	2	2520	6.19 (5.27–7.18)	NA	NA
Lake	8	4858	4.66 (3.49–5.99)	77.19	<0.001
Central	1	1004	4.38 (3.28–5.83)	NA	NA
Zanzibar	1	120	1.67 (0.46–5.87)	NA	NA
Year of study					
1991–2001	5	2980	6.27 (4.22–9.73)	87.93	<0.001
2002–2012	9	23505	5.97 (4.94–7.08)	76.95	<0.001
2013–2023	17	11503	7.48 (3.96–11.97)	98.39	<0.001
Diagnosis method					
ELISA	14	8457	7.79 (4.08–12.53)	97.98	<0.001
RDT	17	29531	6.24 (4.65–6.62)	95.63	<0.001
Study group: general population					
Blood donors	2	3428	18.72 (17.43–20.05)	0	<0.001
Community	3	2977	8.76 (4.55–14.15)	91.17	<0.001
PLWHIV	7	22665	7.31 (5.22–9.72)	93.50	<0.001
Healthcare workers	3	1108	7.06 (4.63–9.93)	56.42	0.10
PWID	2	472	5.32 (3.44–7.57)	0	<0.001
Pregnant women	7	3592	4.72 (3.42–6.21)	62.23	0.02
Others	7	3746	4.18 (2.81–5.79)	0	<0.001

NA: not applicable; CI: confidence interval; ELISA: enzyme-linked immunosorbent assay; RDT: rapid diagnostic test; PLWHIV: people living with HIV; PWID: people who inject drugs.

## Data Availability

The data supporting the findings of this study are available within the article and the supplemental files.

## Abbreviations

CHB: Chronic hepatitis B
CI: Confidence interval
ELISA: Enzyme-linked immunosorbent assay
HBsAg: Hepatitis B surface antigen
HBV: Hepatitis B virus
DNA: Deoxyribonucleic acid
HCW: Healthcare worker
HCC: Hepatocellular carcinoma
MESH: Medical subject heading
PLWHIV: People living with HIV
PRISMA: Preferred Reporting Items for Systematic Reviews and Meta-Analyses
PROSPERO: International Prospective Register of Systematic Reviews
PWID: People who inject drugs
RDT: Rapid diagnostic test.

## Supplementary Materials

Supplementary Materials Supplementary Table 1: risk of bias assessment for selected studies.
