# Supplementary material for: Prevalence of Hepatitis B Virus Infection in Tanzania: A Systematic Review and Meta-Analysis
Source: J Trop Med. 2024 Jun 5;2024:4178240. doi: 10.1155/2024/4178240 (PMC11222000; doi:10.1155/2024/4178240)
Supplement: Supplementary Materials — Supplementary Table 1: risk of bias assessment for selected studies. [file 4178240.f1.pdf]

Supplementary table 1: Risk of bias assessment for selected studies

Study author	Standardized methods for confirming diagnosis	Large enough sample size	Multi-center study	Appropriate statistical methods that report outcomes	Account for confounders	Clear methodology of selection of participants	Population representation
Pellizzer et al [13]							
Stark et al [14]							
Matee et al [15]							
Kilonzo et al [16]							
Miller et al [17]							
Msuya et al [18]							
Rashid et al [19]							
Mirambo et al [20]							
Froeschl et al [21]							
Machange et al [22]							
Valerian et al [23]							
Christian et al [24]							
Meschi et al [25]							
Ramírez-Mena et al [26]							
Kamenya et al [27]							
Kapinga et al [28]							
Muro et al [29]							
Hawkins et al [30]							
Mueller et al [31]							
Shao et al [32]							
Geffert et al [8]							
Shayo et al [33]							

Manyahi et al [34]							
Ng’wamkai et al [35]							
Kawambwa et al [36]							
Mirambo et al [37]							
Shedura et al [38]							
Franzeck et al [39]							
Kilonzo et al [40]							
Menendez et al [41]							

Green cells: low risk; Gray cells: unclear risk; Red cells: high risk
